# A lifetime of neurogenesis in the olfactory system

**DOI:** 10.3389/fnins.2014.00182

**Published:** 2014-06-26

**Authors:** Jessica H. Brann, Stuart J. Firestein

**Affiliations:** ^1^Department of Biology, Loyola University ChicagoChicago, IL, USA; ^2^Department of Biological Sciences, Columbia UniversityNew York, NY, USA; ^3^Department of Neuroscience, Columbia UniversityNew York, NY, USA

**Keywords:** stem cell, regeneration, renewal, aging, proliferation

## Abstract

Neurogenesis continues well beyond embryonic and early postnatal ages in three areas of the nervous system. The subgranular zone supplies new neurons to the dentate gyrus of the hippocampus. The subventricular zone supplies new interneurons to the olfactory bulb, and the olfactory neuroepithelia generate new excitatory sensory neurons that send their axons to the olfactory bulb. The latter two areas are of particular interest as they contribute new neurons to both ends of a first-level circuit governing olfactory perception. The vomeronasal organ and the main olfactory epithelium comprise the primary peripheral olfactory epithelia. These anatomically distinct areas share common features, as each exhibits extensive neurogenesis well beyond the juvenile phase of development. Here we will discuss the effect of age on the structural and functional significance of neurogenesis in the vomeronasal and olfactory epithelia, from juvenile to advanced adult ages, in several common model systems. We will next discuss how age affects the regenerative capacity of these neural stem cells in response to injury. Finally, we will consider the integration of newborn neurons into an existing circuit as it is modified by the age of the animal.

## Introduction

Neurogenesis was initially thought to be restricted to embryonic and early postnatal stages in vertebrates. However, the work of Altman (1962), Kaplan and Hinds (1977), and Graziadei (Graziadei and Graziadei, 1979a,b) clearly demonstrated that neurogenesis is not limited to embryonic development, but continues in specific regions at a significant rate into adulthood. Today, we recognize that neurogenesis is also subject to the mechanisms that govern aging. Neurogenesis occurs in three primary areas in the nervous system. These areas include: the subgranular zone, which supplies new granule cells to the dentate gyrus of the hippocampus; the subventricular zone (SVZ), which supplies new interneurons to the olfactory bulb; and the olfactory neuroepithelia, which generate new excitatory sensory neurons that send their axons to the olfactory bulb. The SVZ and olfactory epithelia are two areas of particular interest as they contribute new neurons to both ends of a first-level circuit governing olfactory perception. Due to space constraints, we have chosen to exclude olfactory ensheathing cells and the rostral migratory stream/subventricular zone from our discussion, all of which have been considered in depth in recent reviews (Whitman and Greer, 2009; Mackay-Sim and St John, 2011; Mobley et al., 2013). We have also restricted ourselves to vertebrate systems here in order to maintain a reasonable focus. Invertebrate systems are often quite different and are well reviewed elsewhere (Cayre et al., 2007; Schmidt, 2007; Faith Kim et al., 2013).

The vomeronasal organ (VNO) and the main olfactory epithelium (OE) comprise the primary peripheral olfactory epithelia. We are beginning to understand the mechanisms by which neurogenesis is controlled in these areas, but many have yet to be clearly defined, perhaps because those that govern embryonic, juvenile, and adult neurogenesis are overlapping but not identical. In addition, the control of early growth, patterning, and differentiation of neurons could be distinct from those found in a regenerating population, and this may well be affected by age. Interestingly, neurogenesis in the olfactory epithelia is rarely accompanied by tumor formation (Bailey and Barton, 1975), implying that this regenerative capacity is in fact carefully regulated.

**Figure 1:**
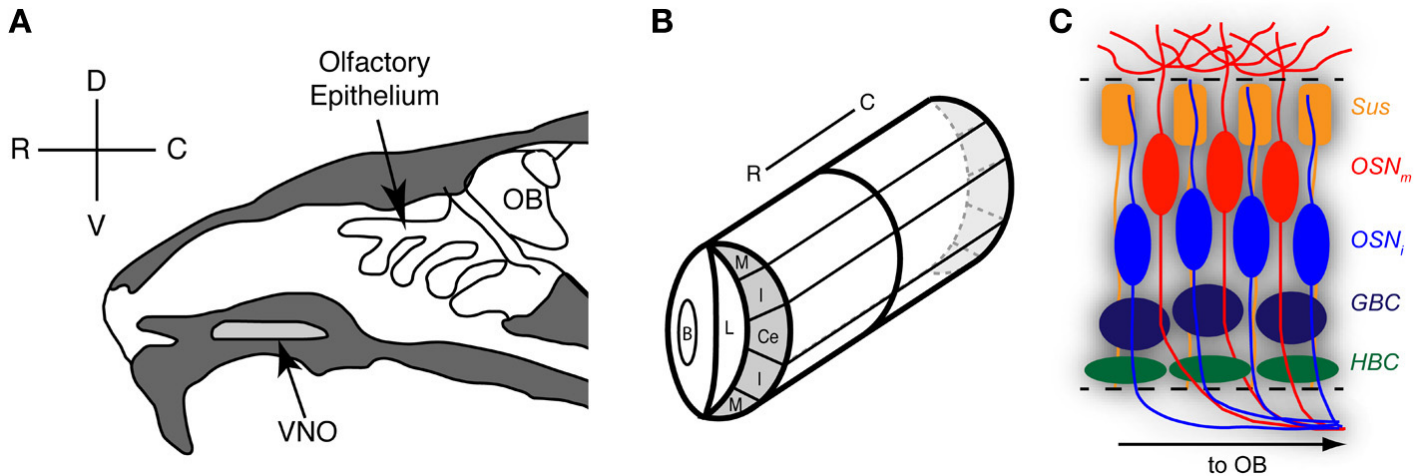
**Organization and zones of the mouse olfactory epithelium**. **(A)** Sagittal schematic of the rodent nose depicting the locations of the olfactory epithelium and the vomeronasal organ (VNO). **(B)** The VNO is a bilaterally symmetrical tubular structure; shown here is one half of a coronal plane as it would appear through the depth of this structure. The marginal zones (M) are found at the extreme dorsal (D) and ventral (V) regions of the VNO. Adjacent to the marginal zones are the intermediate zones (I). In between the two intermediate zones is the central zone (Ce). OB, Olfactory bulb; OE, olfactory epithelium; B, blood vessel; C, caudal; L, lumen; R, rostral. Reprinted with permission from the *Journal of Neuroscience* (Brann and Firestein, 2010). **(C)** The olfactory epithelia are composed of five primary cell types, including the horizontal basal cell (HBC), globose basal cell (GBC), immature olfactory sensory neuron (OSN_i_), mature olfactory sensory neuron (OSN_m_), and sustentacular cell (Sus).

Stem cells resident in the olfactory epithelia generate sensory neurons throughout the life of the animal (Brann and Firestein, 2010; Kondo et al., 2010). These sensory neurons, while specialized for transducing chemical stimuli, are indeed true neurons (not specialized epithelial cells) of the Golgi type I, possessing a long axon forming glutamatergic synapses with mitral cells in the olfactory bulb (OB) (Firestein, 2001). The VNO and OE (Figure 1) share many anatomical and functional features. They are both pseudostratified columnar epithelia composed of basal cells, immature, and mature sensory neurons, Bowman's gland cells, and sustentacular (supporting) cells. Mature sensory neurons are bipolar neurons, with an elongated dendrite and elaborate cilia in which odor detection and transduction takes place.

The vomeronasal epithelium is a wide neuroepithelium and is found within a paired bony capsule (the vomeronasal bone) at the base of the anterior septum in the nose; this structure is typically termed the VNO. An autonomically controlled vascular pump governs stimulus access to the VNO in rodents (Meredith et al., 1980). While historically associated with pheromone detection, the VNO appears to be functionally restricted to the detection of non-volatile odorants (Garrosa et al., 1998) and has been well established as a primary detector for odor information concerning social organization and reproductive status, including pheromones (Mombaerts, 2006). In addition, in reptilian species such as the turtle, lizard, and garter snake, the VNO also detects prey items (Fadool et al., 2001). Odor detection in the sensory neuron occurs via a large family of G protein-coupled receptors (GPCRs) termed vomeronasal receptors (VRs) (Dulac and Axel, 1995; Herrada and Dulac, 1997; Matsunami and Buck, 1997; Ryba and Tirindelli, 1997). There are two distinct populations of vomeronasal sensory neurons (VSNs) characterized by receptor and G-protein expression. Apically situated neurons express Gαi2 and V1Rs and project to the anterior accessory OB while basal neurons express Gαo and V2Rs and project to the posterior accessory OB (Mombaerts, 2004).

The main OE is found posterior to the VNO in the adult nasal cavity, and covers elaborate cartilaginous turbinates, structures that serve to increase surface area for greater stimulus access (Figure 1). The main OE is structurally similar to the VNO in that the cell types are conserved; however, there do not appear to be layers of mature neurons such as the apical/basal pattern of VSNs. Sensory neurons in the OE express odorant receptors (ORs); in rodents, the family of ORs is quite large (~1400 different genes) (Zhang et al., 2007). Each mature sensory neuron expresses G_olf_, olfactory marker protein (OMP), neural cell adhesion molecule (NCAM; Schwob et al., 1994), and projects to a single glomerulus in the OB (Mombaerts, 2006).

After an odorant is detected by peripheral sensory neurons, the signal is relayed to the OB in the brain. This portion of the olfactory system, the OB, is also subject to modification by lifelong neurogenesis. The SVZ lines the lateral ventricles and generates neurons that migrate via the rostral migratory stream to yield two cell types, namely the periglomerular and granule cells of the OB. The function of SVZ neurogenesis is unclear, but may contribute to cellular plasticity necessary for organisms to adapt to environmental change (Lepousez et al., 2013). For the purposes of this review, we will not consider neurogenesis in this portion of the olfactory system, as the cell types generated are inhibitory and not related to those generated peripherally throughout the life of the animal.

The stem cells in the olfactory epithelia are capable of generating neurons as well as supporting (glial) cells (Leung et al., 2007). The neurons that are generated choose a receptor “identity”; each sensory neuron usually expresses a single vomeronasal or odorant receptor, and this gene choice is highly regulated (Shykind et al., 2004; Lomvardas et al., 2006; Magklara et al., 2011; Lyons et al., 2013). In addition, the developmental process of aging regulates olfactory neurogenesis. Hence neurogenesis in these tissues is a complex phenomenon governed by a series of molecular regulatory mechanisms.

When one considers the word “age” in the context of neurogenesis, there are two interpretations; organismal age, and age of the progenitor or stem cell. The former is relatively easily defined, but the latter is more difficult to describe. In this review, we will discuss the effects of organismal age on neurogenesis, the effect of organismal age on the regenerative capacity of neurogenesis, and how organismal age may impact the incorporation of new neurons into existing circuits in the two peripheral olfactory epithelia.

## The embryonic origins of the olfactory epithelia

The rodent VNO and OE arise from the olfactory placode or pit, the invagination of which forms the nasal cavities early in development (Suarez et al., 2012). In the murine OE, the first signs of cellular differentiation can be observed at embryonic day 10 (E10), when the epithelium already contains dark (embryonic stem cells; Pax7+; Murdoch et al., 2010) and pale (developing sensory neurons) cells. At this stage, the proliferation of progenitor cells is dependent upon retinoic acid and *Six1* (Ikeda et al., 2010; Paschaki et al., 2013). However, the hallmark layering of the epithelium is not visible until later in development (E13–E15). Before this stage, the elongated nuclei of stem cells are present in both the apical and basal compartments; afterward, the stem cells become restricted to the basal compartment, and the apical layer terminally divides to become the sustentacular cells. This transition is marked by Fezf2 (a zinc finger transcription factor) restriction to the sustentacular layer in the VNO (Eckler et al., 2011). Insm1, also a zinc-finger transcription factor, is transiently expressed by progenitors at this stage and may promote this transition (Rosenbaum et al., 2011). Dendrites of the sensory neurons are first visible at E11, and this is coincident with first contact of sensory axons with the OB, successful penetration of which is dependent upon Neurog1/2 (Shaker et al., 2012). However, functionality is not necessarily implied by anatomy at this stage, as the underlying vasculature in the basal lamina and Bowman's glands are not present until E15 and E17, respectively (Cuschieri and Bannister, 1975). As Cuschieri and Bannister (1975) point out, it is interesting to note that the nuclei of the embryonic stem cells differ from that of the “differentiated” basal cells found in the early postnatal and adult mouse. Their conclusion was that perhaps stem cell capacities were not conserved between the two populations; indeed, they no longer express Pax7 (Murdoch et al., 2010) or contain nestin-expressing radial glia-like progenitors (Murdoch and Roskams, 2008), both of which which may indicate a loss of embryonic pluripotency.

In general, the development of the murine VNO is similar, although delayed relative to that of the OE. Following placode invagination, a recess in the medial wall forms the VNO at E11. By E13, clear mitoses are restricted in the basal layer of the vomeronasal epithelium. During this time, Notch1-expressing cells are found throughout the VNO but the expression of Notch1 decreases with development (Wakabayashi and Ichikawa, 2007). This is consistent with the function of Notch promoting differentiation of progenitor cells in other neurogenic systems. By E19 in the rat (shortly before birth), however, few mature neurons (as indicated by OMP expression) are observed (Matsuoka et al., 2002). These data, in combination with observations that architectural, histochemical, and ultrastructural features of immaturity are still observed at birth (Garrosa et al., 1998; Taniguchi, 2008) indicate the rodent vomeronasal epithelium differentiates more slowly than the main OE. By the end of the third postnatal week, the rat VNO is morphologically mature (Garrosa and Coca, 1991).

The structural development of the vomeronasal epithelium in the garter snake (*Thamnophis sirtalis*) appears to be more similar to that of the OE, but by birth the neuronal precursors are restricted to the basal layer (Holtzman, 1998). In the frog *Rana japonica*, late VNO development relative to OE formation is also observed; the OE is largely adult-like in tadpoles 1 month after hatching, but the VNO is not complete until the end of metamorphosis (Taniguchi et al., 1996). Late VNO development is also seen in the opossum, *Monodelphis domestica*, although this marsupial is particularly interesting because the VNO is in an extreme state of immaturity at birth and provides an opportunity to examine embryonic-like processes in an early postnatal animal (Couper Leo and Brunjes, 1999). Together, these results suggest the OE is functional earlier in development than the vomeronasal epithelium in most vertebrate species.

## On the identity of the adult olfactory neural stem cell

From late embryonic to postnatal stages, basal cells are thought to be responsible for generating sensory neurons. The basal cell population gives rise to Ascl1+ progenitors and subsequently Neurogenin-1 and NeuroD1+ immediate neuronal precursors (Packard et al., 2011a; Suarez et al., 2012). Following this stage, GAP-43+ immature neurons terminally differentiate into OMP-expressing mature neurons. This lineage is conserved in both the VNO and OE. However, in general more is known about progenitor cell activation in the OE than in the VNO.

Two populations of basal progenitor cells are found in juvenile and adult olfactory epithelia, including horizontal basal cells (HBC) and globose basal cells (GBC). The identity of a single juvenile or adult stem cell population remains contentious. Confounding the matter is the fact that the GBCs in the adult are similar but may not be identical to the embryonic progenitors, and the HBCs appear in late embryogenesis due in part to Ascl1, and whose activation is dependent upon ΔNp63 (Fletcher et al., 2011; Packard et al., 2011b; Krolewski et al., 2012). Clearly, the GBC can give rise to all cell types in the OE (Schwartz Levey et al., 1991; Caggiano et al., 1994; Huard et al., 1998; Jang et al., 2003; Beites et al., 2005; Schwob and Jang, 2006). Recent work using powerful genetic tools to perform lineage tracing has demonstrated that the HBC can also generate all cell types found in the OE and is also a neuronal stem cell (Duggan and Ngai, 2007; Leung et al., 2007; Iwai et al., 2008; Mackay-Sim, 2010). Wnt signaling regulates the activation of both GBCs and HBCs (Sox2+) in early postnatal mouse OE; furthermore, Wnt signaling is required for recovery following chemical lesion in adult mice (age not stated; Wang et al., 2011b).

Neurogenesis in the olfactory epithelia comes in two flavors: that which is required for ongoing regeneration in an intact epithelium, and that which is required following injury. GBCs are likely the progenitor for many of the neurons made during ongoing neurogenesis and during reconstitution following a mild injury; most cycle rapidly (Huard and Schwob, 1995) and incorporate a marker of DNA synthesis, 5-bromo-2′-deoxyuridine (BrdU) at a high rate. Recently however a subpopulation of label-retaining GBCs were shown to cycle slowly, a characteristic previously demonstrated only in HBCs (Jang et al., 2014) and one that is common to adult stem cells in other tissues (Fletcher et al., 2011). HBCs are immunoreactive for cytokeratins (K5/K14; Holbrook et al., 1995; Comte et al., 2004), incorporate BrdU to a limited degree, divide at a slow rate (Mackay-Sim and Kittel, 1991a), and can be considered quiescent neural stem cells (after Wang et al., 2011a) that respond to severe injury. The HBC also has a conserved adhesion receptor expression profile similar to other stem cells (Carter et al., 2004). However, there remains a disagreement concerning the role of the HBC in ongoing neuronal turnover and mature neuron-specific injury (via bulbectomy or the removal of the target of the sensory neurons) vs. its role in neurogenesis following a severe chemical lesion (commonly made with methyl bromide or methimazole) that damages all cell types of the epithelia. Using fate-mapping analysis via an inducible Krt5-cre in combination with a LacZ reporter line, Leung et al. found that a severe lesion that disrupts the integrity of the epithelium is required to recruit HBCs. A neuron-specific lesion, namely olfactory bulbectomy (OBX), did not recruit HBCs (Leung et al., 2007) in their experiments. In direct conflict with the results of Leung et al. another group found that both normal neuronal turnover and OBX recruited HBC activity when using a constitutively active Krt5-cre strain (Iwai et al., 2008). Future work may clarify these particular results. Here, we conclude that there are likely two populations of multipotent stem cells competent to generate neurons in the olfactory epithelia, including both the horizontal and GBC. However, we would point out that the majority of this work was completed in young animals, and hence we do not know if the same signaling mechanisms govern the neurogenic process in aged animals.

## The effect of organismal age on neurogenesis in the olfactory epithelia

The occurrence of neurogenesis in the olfactory epithelia of vertebrates has been well documented for over 50 years. Basal cells in the VNO and OE clearly retain the capacity to generate new neurons throughout life. The genetic and molecular determinants of neurogenesis in the olfactory epithelia appear to be largely conserved between embryonic stages and postnatal stages. Interestingly, this same process is also conserved amongst epithelia capable of regeneration (auditory and visual, for example; for a recent extensive review please see Bermingham-McDonogh and Reh, 2011).

It has been suggested that the neurogenesis observed in the olfactory epithelia is due to turnover of the population of immature sensory neurons, rather than due to replenishment of mature sensory neurons (Hinds et al., 1984; Mackay-Sim and Kittel, 1991b). This implies newborn neurons do not in fact reach a mature state. However, by 30 days after BrdU labeling in all ages tested (1–24 months of age), a proportion of BrdU-labeled cells will also express OMP, a marker of neuronal maturity, indicating newborn neurons do indeed become mature neurons (Brann and Firestein, 2010). Whether newborn olfactory sensory neurons form proper synaptic connections in the OB is a question discussed below; however, retrograde labeling of newborn VSNs suggests their axons are indeed able to reach the AOB (Barber, 1981a). Regardless, we do know that the stem cells in the VNO and OE are capable of reconstituting the epithelia following a lesion (discussed below) and hence the process of neurogenesis is assumed to be functional in an unlesioned animal as well.

**Figure 2:**
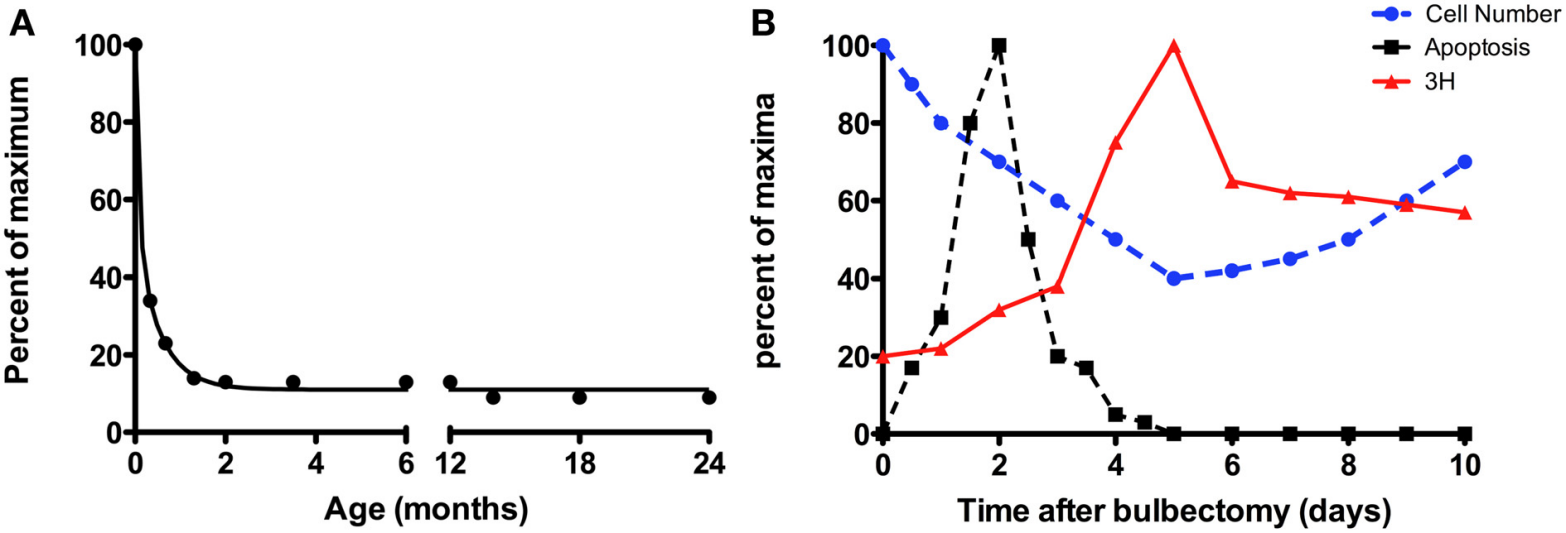
**The effect of organismal age on the rate of neurogenesis**. **(A)** The number of cells incorporating BrdU per mm in the rodent declines precipitously over the first month of life, but reaches a steady-state level in the adult that does not appear to be profoundly affected by aging. Graph of the authors' calculations from Wilson and Raisman (1980); Weiler et al. (1999); Weiler (2005); Brann and Firestein (2010), normalized to peak value reported. **(B)** The neurogenic response of the OE following lesion (bulbectomy). Figure is adapted from Kastner et al. (2000). Cell number (blue dashed line, filled circles), number of apoptotic cells (black dashed line, filled squares), and cells incorporating ^3^H-thymidine (red solid line, triangles) in the olfactory epithelium of mice expressed relative to their respective maxima. The levels of mitotic cells (red) peak 5 days post-surgery, while total cell number (dotted line) is at its lowest following surgery.

The bulk of the studies examining neurogenesis thus far have been done in young adults, not aged adults (24–30 months of age for mice). More recently we have begun to investigate the role of organismal age in regulating proliferation in the olfactory epithelia. In the VNO, basal progenitor cells are capable of extensive neurogenesis although many newborn cells die before becoming functional neurons (Martinez-Marcos et al., 2005). The majority of neurogenesis in the early postnatal rodent VNO appears to be due to growth related processes rather than neuronal replacement, and is perhaps an extension of development, as the VNO matures later than the OE (as discussed above). The vomeronasal epithelium can be divided into zones; neurogenesis from basal cells in the marginal zones near the dorsal and ventral aspects of the VNO is predominantly responsible for growth, while that in the central zone is associated with neuronal replacement (Barber and Raisman, 1978a; Wilson and Raisman, 1980; Weiler et al., 1999; Giacobini et al., 2000; Martinez-Marcos et al., 2000; Weiler, 2005; De La Rosa-Prieto et al., 2009; Brann and Firestein, 2010). While the level of proliferation in rodents is high at birth, as assessed by either ^3^H-thymidine or BrdU incorporation, these levels fall precipitously over first month of life. However, by sexual maturity, i.e., ~2 months of age through advanced ages (over 20 months of age), proliferation has leveled out to roughly 10% of the level observed at birth (Figure 2A; authors' calculations from (Wilson and Raisman, 1980; Weiler et al., 1999; Weiler, 2005; Brann and Firestein, 2010). This is paralleled by Gγ8 expression, a GTP-binding protein whose function in neurogenesis is unclear, but may signify a switch from the developmentally expressed subunit to a mature G-protein (either Gαo or Gαi2) as newborn neurons mature (Ryba and Tirindelli, 1995). The decline in neurogenesis with age may be due in part to a decline in the number of basal cells (Garrosa and Coca, 1991); indeed Notch1 expression is largely restricted to the marginal zones by 4–5 months of age (Wakabayashi and Ichikawa, 2007). However, there remain a few basal cells in the central zone of the VNO, which can be reactivated by injury (discussed below; Brann and Firestein, 2010). Regardless of the decline in rate with age, neurogenesis yields both V1R and V2R expressing populations in a similar proportion, indicating stochastic production of VSNs (de la Rosa-Prieto et al., 2010). The decline in “normal” proliferative levels is observed in other species, for example the opossum (Jia and Halpern, 1998) and snake (Holtzman, 1998) and is likely to be a feature common to all vertebrates.

In the OE of rodents, continuous neurogenesis also occurs throughout life (Graziadei and Monti Graziadei, 1978; Hinds et al., 1984). Long-lasting neurogenesis occurs in humans as well; when OE was isolated from middle aged and elderly postmortem OE and grown *in vitro*, some newborn cells did express OMP, albeit at a low rate (Hahn et al., 2005). However, as in the VNO, the rate of proliferation in the OE also declines with age [mouse (Loo et al., 1996); guinea pig (Nakamura et al., 1998); and dog (Hirai et al., 1996)]. Similar to the VNO, during the first year of life in the rat OE, neurogenesis was shown to contribute largely to growth rather than replacement (Weiler and Farbman, 1997). That is, the OE continued to grow by adding new cells for up to 1 year postnatal, i.e., well into middle age. Proliferation decreased dramatically over early postnatal ages (from 151 cells/mm at P1, postnatal day 1, to 24 cells/mm at 3 months of age) to a low of 8 cells/mm at 1 year of age. This occurred as the total surface area of the rat MOE increased with age (Weiler and Farbman, 1997, 1998a). During the same period, the proliferation of supporting cells also declined with age. At early postnatal ages (P1, postnatal day 1) the proliferation rate was high (80 cells/mm) but declined quickly to 12 cells/mm by P21, and even further by 1 year of age to 0.4 cells/mm (Weiler and Farbman, 1998b). The decline in proliferation parallels the decline in apoptosis (Mackay-Sim and Kittel, 1991a; Fung et al., 1997; Kondo et al., 2010) as well as the decline in Ascl1, a proneural gene required for the generation of olfactory sensory neurons, with advancing age (Guillemot et al., 1993; Cau et al., 2002; Watanabe et al., 2007). However, the time required to generate a new neuron, from birth to maturation, is similar at all ages (Kondo et al., 2010). Advanced age ultimately is associated with deficits in epidermal growth factor signaling in the OE (Enwere et al., 2004), decreased olfactory sensitivity, and impaired olfactory discrimination learning [mice (Fantana et al., 2008); rat (Schoenbaum et al., 2002); primate (Aujard and Nemoz-Bertholet, 2004); human (Doty and Kamath, 2014)], a curious outcome for a tissue capable of regeneration.

Not only is the rate of proliferation regulated by aging, but the end result of neurogenesis appears to be as well. The gene expression profile of odorant and VRs has been observed to change from early postnatal to advanced ages (Zhang and Firestein, 2002; Zhang et al., 2004, 2010; Lee et al., 2009; Rodriguez-Gil et al., 2010), such that receptor gene expression is turned on and off at different ages during the lifetime of the animal. However, this work has recently been challenged (Khan et al., 2013) using new NanoString technology, so future work is needed to clarify these patterns. In addition, while the lifespan of an OSN in young adult animals is generally reported to be approximately 30 days (Graziadei and Graziadei, 1979b), other experiments indicate that OSN lifespan may be as long as 90 days (Wilson and Raisman, 1980; Mackay-Sim and Kittel, 1991b) or even a year (Hinds et al., 1984). Recent evidence supports the idea that neuronal turnover in aged animals is even lower than that in young animals, and therefore the life a mature OSN may be even longer as animals age (Kondo et al., 2010).

The cell cycle of active proliferative cells in the OE has been estimated to be 17 h (Huard and Schwob, 1995). However, there is evidence that this too is regulated by aging; in juvenile guinea pigs, the rate of division was measured to be faster than that observed in adults (Higuchi et al., 2005). Interestingly, the regulation of cell cycle genes was particularly prominent in a common aging model, the senescence-accelerated mouse (SAM) when examined by microarray analysis (Getchell et al., 2003). In summary, it is now well established that neurogenesis continues into adulthood in the VNO and OE but slows with age. Aging may ultimately disrupt the structure of the epithelia (Rosli et al., 1999) and the expression of regulators of the cell cycle (Legrier et al., 2001) in the OE, but perhaps a larger question is that of the “age” of the stem cell itself, and whether the regenerative capacity of this stem cell is regulated by developmental stage or advanced age.

## The effect of organismal age on the regenerative capacity of neurogenesis in the olfactory epithelia

The regenerative capacity of the vomeronasal and olfactory epithelia has been probed quite extensively with several lesion paradigms. Some of these methods selectively target the mature neuronal population, such as removal of the OB (OBX) or severing the olfactory nerve (axotomy or nerve transection). Both of these techniques result in the initial degeneration of sensory neurons, followed by a massive upregulation of proliferation of mitotic progenitor cells. Other perturbations include sensory deprivation (naris occlusion) and olfactotoxicants (chemical ablation). Methyl bromide gas or methimazole are examples of chemical ablation; these typically evoke the most severe lesions, resulting in the loss not only of sensory neurons but other cell types (supporting, GBC) as well. More recently, genetically mediated lesion techniques have proven useful in dissecting the neurogenic process (Chen et al., 2005).

The literature on recovery following lesion in the VNO is relatively sparse. In the rodent VNO, the robust regenerative capacity of the stem cell population was demonstrated by Barber and Raisman (1978a,b); sensory neurons degraded within 8 days, and proliferation was significantly increased when examined 10–20 days following nerve transection or removal of the accessory OB (Barber and Raisman, 1978b). These results were verified in rat (Yoshida-Matsuoka et al., 2000), mouse (Wakabayashi and Ichikawa, 2007), hamster (Ichikawa et al., 1998), opossum (Jia and Halpern, 1998), and garter snake (Wang and Halpern, 1988). However, most of this work was performed primarily in young animals (or the age of the animals is not explicitly stated and may be assumed to be in young animals). Recently, we have shown that the regenerative response following injury (in this case, OBX) is considerable, even out to advanced ages (24 months of age in mice; (Brann and Firestein, 2010), implying the stem cell found in aged mice is quite capable of undergoing extensive neurogenesis. In addition, seasonal additions to the VNO such as those seen in lizards and salamanders support the idea that these stem cells have a resilient regenerative capacity (Dawley et al., 2006; Delgado-Gonzalez et al., 2011). An unanswered question concerns whether recovery from a lesion to the VNO is incomplete (Ichikawa et al., 1998); however, in the case of bulbectomy studies, this is reasonable as new neurons send their axons to the now absent target, die, and another wave of neurogenesis is triggered.

The regenerative capacity of the OE has by comparison been investigated much more thoroughly. In most reported techniques, anatomical (Schwob et al., 1995) and functional (Blanco-Hernandez et al., 2012) recovery is evident within ~45 days and odorant receptor expression patterns are reestablished within 90 days (Iwema et al., 2004). Chemical ablation of the OE (by compounds such as zinc sulfate, methyl bromide, and methimazole) or lesion following removal of the OB is followed by rapid proliferation of basal cells (Matulionis, 1975; Hurtt et al., 1988; Genter et al., 1995, 1996; Williams et al., 2004) producing more than 8–10 million new neurons in total (Carter et al., 2004; Suarez et al., 2012), demonstrating how remarkably robust neurogenesis can be in these epithelia. In lesioned rodent epithelia, degeneration occurs quickly (Figure 2B) via apoptotic cell death, followed by a steep increase in proliferation. Mature neurons (as indicated by OMP expression) are visible 8–10 days after lesion.

There have been several demonstrations that the olfactory epithelia, when damaged by lesion techniques that do spur neurogenesis, often fail to completely recover as measured by cell density and epithelial thickness (Costanzo and Graziadei, 1983; Schwartz Levey et al., 1991; Suzukawa et al., 2011). These results raise the possibility of a limit to the regenerative capacity of the neural stem cell, perhaps a phenomenon aging may exacerbate. However, while we know much about cell dynamics of the regenerative process in early postnatal and young adult animals [mice (Suzuki et al., 1998); teleost fish (Bettini et al., 2006); primates (Graziadei et al., 1980)], we do not currently know much about how the aged epithelium contends with regeneration following injury. Indeed, recovery from zinc sulfate lesion was less efficient in adult (6 month old) mice than in young (1 month old) mice (Ducray et al., 2002a), but functional recovery occurred even in the adult group (Ducray et al., 2002b). However, chemical lesions are not equivalent in the amount of damage they cause (Bergman et al., 2002). Using a different lesion method (3,3′-iminodipropionitrile; IDPN), Genter and Ali (1998) demonstrated that there is an age-related susceptibility to damage caused by IDPN, perhaps confounding the interpretation of chemical lesion results in aged mice (Bovetti et al., 2011). Recent work by Suzukawa et al. (2011) investigated the efficacy with which the aged OE responded to methimazole-induced lesion and found that the numbers of proliferative cells in aged (16 month old) animals post-lesion were much lower than either their 3 month or 10 day old counterparts, the number of immature neurons were lower, and ultimately, the number of mature neurons (OMP expressing) in aged mice were approximately a third of that observed in the young groups (Suzukawa et al., 2011).

## The effect of organismal age on the integration new neurons into existing circuits

Neurogenesis and regeneration is a complex task for a sensory system to accomplish, particularly in the case of the olfactory system. This system has not one, but two components continually subject to modification by neurogenesis. How is a sensory percept maintained in the face of such plasticity? Clearly communication occurs between the components of the olfactory system regarding the status of neurogenic activity. For example, the function of mitral cells in the OB affects the number of sensory neurons surviving in the epithelium (Weiler and Farbman, 1999; Cavallin et al., 2010), while ablation of the OB not only modulates neurogenesis in the OE, but also causes cell death in the piriform cortex, the target of mitral cell axons (Leung and Wilson, 2003).

Neurogenesis is a multistep process; the generation of new neurons from progenitor populations not only requires sequential onset of basic helix loop helix transcription factors such as Sox2, Pax6, and Hes1, but expression of Ascl1, a proneural gene involved in neuronal differentiation. Additionally, the axons of the newborn neurons must successfully reach their targets in the OB, and form appropriate synapses. Two previous studies indicated that VSNs perhaps do not reach the AOB (Barber, 1981b; Matsuoka et al., 2002). However, both of these studies used a nerve transection technique that can cause scarring, leaving open the possibility the growing axons from newborn neurons could not efficiently traverse the damaged tissue. More recently, ~60% of newborn VSN axons in young (2–4 months of age) mice were found to reach the AOB, as assessed by combinatorial BrdU pulse labeling and iontophoretic injections of dextran conjugated tetramethylrhodamine into the AOB (de la Rosa-Prieto et al., 2011). Similar results have been obtained in other species where newborn neurons re-establish connections to the main OB as well [hamster (Costanzo and Graziadei, 1983); mouse (Burd, 1993); rat (Schwob et al., 1999); zebrafish (Iqbal and Byrd-Jacobs, 2010)], although functional recovery may precede morphological recovery (Hurtt et al., 1988).

While these experiments support the idea that newborn neurons can successfully target and integrate into circuits in the OB, whether neurons generated in aged animals are able to accomplish this task is unclear. We also do not know if the process of outgrowth, targeting, and successful synapse formation is different in an intact system vs. a lesioned epithelium. There is some evidence that olfactory sensory neurons are able to accomplish this in the face of a lesion challenge. In hamster, axons from newborn olfactory sensory neurons, generated following OBX in aged animals, are able to reconnect with the OB (Morrison and Costanzo, 1995). This may not hold true for other rodent or other model systems, and it is unknown if more severe lesions would result in a different outcome.

## Conclusions

Understanding the regenerative capacity of the brain throughout its lifespan is an important goal for many neuroscientists in the hopes that the mechanisms governing neurogenesis might be exploited to repair neuronal loss caused by aging, injury, or neurodegenerative disease. The process of neurogenesis therefore understandably fascinates many of us. One conundrum of this process is the “why”—why is neurogenesis necessary? What functions does it fulfill? Is it merely a holdover of a developmental process, or is it necessary for optimal organismal function? While the answers are becoming perhaps more clear regarding the role of SVZ neurogenesis in learning (Mandairon et al., 2011; Moreno et al., 2014), we are only just beginning to understand the role of neurogenesis, and the effects of organismal age upon it, in the olfactory epithelia.

The function, potency, or perhaps the replicative cycles remaining, of the majority of stem cell types, neural or otherwise, typically declines with increasing age (Signer and Morrison, 2013). The aging phenotype is thus likely due to accumulated mutations in addition to compromised stem cell function as an organism ages. This results the loss of neurons, aberrant function, increased neuronal longevity (and additional opportunities for accumulation of mutations), or a lack of regenerative capacity following injury. The stem cells resident in the vomeronasal and olfactory epithelia exhibit a profound resistance to the types of cellular aging observed in other tissues, and provide insight into the regulation of stem cells beyond embryonic or early postnatal stages.

The idea that stem cells are influenced by the environment in which they reside is not new; however, recent developments indicating that the environment might be more influential than previously thought are worthy of consideration. For instance, the neural stem cell in the OE is relatively active when compared to neural stem cells in the remainder of the proliferative postnatal and adult brain; does this indicate that the stem cells in this tissue are under a constant source of stress? What is causing the normally continually proliferative mode of the stem cells in the OE vs. the remainder of the nervous system? What molecular signals cause aged tissues to exhibit less proliferation than young animals, but are clearly altered during regeneration? Genomic and proteomic expression profiling may provide answers. Getchell et al. addressed this by performing gene expression analysis of the OE in the SAM and normal aging mouse, highlighting genes involved in the stress response in particular as being regulated by aging (Getchell et al., 2004; Poon et al., 2005). Recent work by Schwob et al. has identified a cohort of genes whose expression is specific to GBCs in young adult mice; future analysis may reveal whether this transcriptional profile is altered with aging (Krolewski et al., 2013). As we look toward the future, as Graziadei observed many years ago (Graziadei and Monti Graziadei, 1983), studies of the olfactory system's regenerative capacity “could contribute to the understanding of the phenomena related to the control of neurogenesis, plasticity of connections, and target recognition.”

We have discussed neurogenesis throughout the lifespan of vertebrates, considering embryonic, early postnatal, juvenile and aged animals, in a system that undergoes lifelong neurogenesis. A question for future research concerns whether neurogenesis in aged animals is in fact a recapitulation of embryonic mechanisms, or if the stem cell has become restricted in some way. Recent work by Heron et al. (2013) demonstrated that gene expression patterns in young adult mice during neurogenesis following bulbectomy were indeed similar to embryonic processes, but future work is needed to ascertain whether this applies to the aged epithelia, or epithelia recovering from a chemical lesion. A second question is whether there is an overarching homeostatic balance between the numbers of glia and neurons in the VNO and OE. Evidence indicates that the relationship between neurons and glia is reciprocal (Tolbert et al., 2004) and thus may play a role in regulating neurogenesis in these tissues from adult and aged animals. Finally, an interesting future question may concern circadian rhythms, which are disrupted with aging (Goergen et al., 2002; Campos Costa et al., 2013). Circadian rhythms also regulate neurogenesis (Schnell et al., 2014) and OB activity (Granados-Fuentes et al., 2006); hence, a disregulation of clock genes such as Per1 and Per2 may be a source of explanation for the decline in proliferation observed with aging in the olfactory epithelia.

Finally, we would affirm that neurogenesis in the adult olfactory system has a role beyond simple anatomical growth, and is likely a necessary strategy to combat environmental damage. Perhaps of most utility is to recognize the proliferative nature of these neural stem cells so that we may exploit them for their therapeutic potential. After all, one thing is clear; clinical applications may be vast if we pursue a more thorough understanding of the processes regulating this repository of accessible neural stem cells (Schwob and Jang, 2006; Delorme et al., 2010; Wetzig et al., 2011; Mackay-Sim, 2012).

### Conflict of interest statement

The authors declare that the research was conducted in the absence of any commercial or financial relationships that could be construed as a potential conflict of interest.
